# A novel seasonal-spatial integrated model for improving the economic-environmental performance of crop production

**DOI:** 10.1016/j.mex.2022.101906

**Published:** 2022-11-07

**Authors:** Man Li, Zhe Guo, Wei Zhang

**Affiliations:** a Department of Applied Economics, Utah State University, Logan, UT 84322, United States; b International Food Policy Research Institute, Washington, DC 20005, United States

**Keywords:** Seasonal-spatial optimization, Economic-ecological integrated modeling, Cropland use planning, InVEST model, Crop production, Fertilizer application, Nitrogen runoff, Bangladesh

## Abstract

Excess agricultural nitrogen, mainly from manure and chemical fertilizers, is a primary source of nutrient pollution and presents serious environmental threats to natural ecosystems and human health. Improvements in nitrogen-use efficiency in crop production are critical for addressing the triple challenge of food insecurity, environmental degradation, and climate change. Approaches such as sustainable intensification that stress technological innovations have received the most attention. But science-based cropland use planning, a promising complementary approach, has so far been largely overlooked. Here we develop a spatially integrated economic-ecological modeling method to assess this previously unexplored potential for improving the economic-environmental performance of crop production by examining the seasonal and spatial implications of cropland and fertilizer use in Bangladesh. In doing so, we aim to make the modeling method accessible to researchers and practitioners interested in achieving the dual goal of food production and environmental sustainability for countries that are characterized by seasonal and spatial variations in crop mix and cropping practices.

- The modeling method combines economic and ecological models to explore trade-offs between food security and environmental sustainability.
- The modeling method considers both spatial and seasonal dimensions when measuring trade-offs.
- The modeling method is highly suitable for adaptation to study topics concerning food security-ecosystem service trade-offs or to inform the design of related models.

## Graphical abstract

**Figure:**
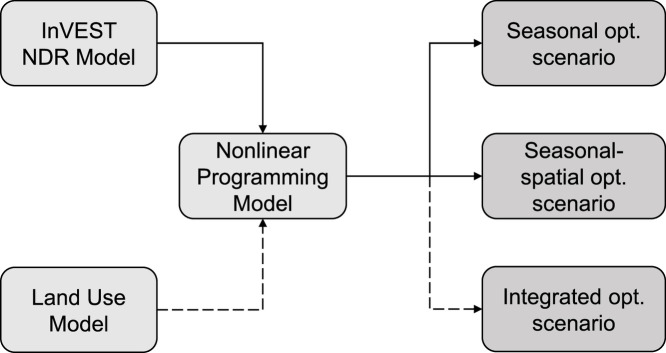

Specifications table

**Table:** 

Subject area:	Environmental Science
More specific subject area:	*Integrated economic-ecological modeling*
Name of your method:	*Seasonal-spatial integrated modeling*
Name and reference of original method:	*NA*
Resource availability:	*NDR model is available from*https://naturalcapitalproject.stanford.edu/software/invest

## Method details

**Fig. 1:**
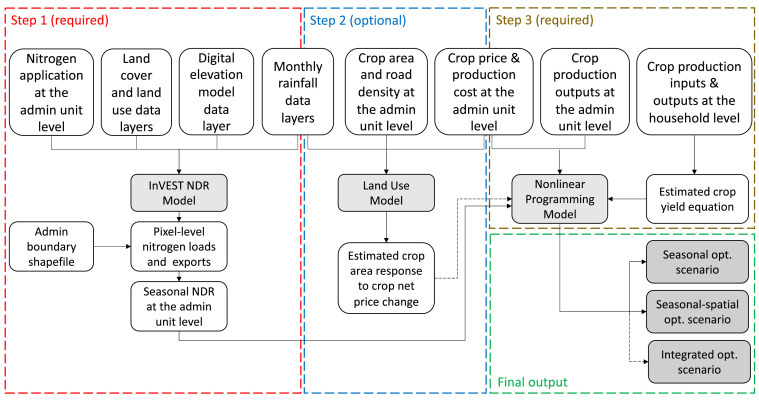
Workflow of the seasonal-spatial integrated model. Note: The black dashed arrow line from Step 2 to Step 3 indicates that the process is *optiona*l; the black dashed arrow line in Final output block indicates that the integrated optimization scenario is *optional*.

In this paper, we develop a spatially integrated economic-ecological modeling method. While the model was originally developed to examine the production of rice and non-rice crops and the impact of fertilizer use across three cropping seasons in Bangladesh [1], the modeling method is highly suitable for adaptation to study topics concerning food security-ecosystem service trade-offs or to inform the design of related models. The version introduced here places particular emphasis on trade-offs between crop production and nitrogen fertilizer-generated nutrient pollution by examining the seasonal and spatial implications of cropland and fertilizer use. With proper adaptations, the modeling method can be used to study trade-offs between food and nutrition security and the provision of other ecosystem services such as carbon sequestration or sediment retention. The modeling framework consists of three models (Fig. 1). The first one is a spatially explicit nutrient delivery ratio (NDR) model for assessing the effect of cropping intensity and fertilizer application rate on the disservice of nutrient runoff [2], [3], [4] (see Step 1 block in Fig. 1). The second model is an econometric land use model for empirically examining how cultivation area responds to the price change of each of the two main crop groups (rice and non-rice crops) for each of the three cropping seasons—spring, summer, and winter (see Step 2 block in Fig. 1). The third model is a nonlinear programming model for minimizing total nitrogen loss in the country from soil across the annual three crop growing seasons through changing nitrogen application rate and reallocating cultivation between rice and non-rice crops across seasons (and districts), subject to a set of economic and physical constraints concerning total annual output value, food security, and season-specific cropland availability (see Step 3 block in Fig. 1).

We devise three optimization scenarios (see Final output block in Fig. 1):

- Seasonal optimization: Fertilizer application and crop cultivation area are optimized across seasonal variations within each administrative unit such as district, while maintaining farmers’ production revenue in districts.
- Seasonal-spatial optimization: Fertilizer application and crop cultivation area are optimized across seasons and districts within the country, while maintaining that the country's farmers as a whole would not see a loss in production revenue.
- Integrated optimization: Fertilizer application and crop price are optimized across seasons and districts within the country, with the help of price instruments that guide the reallocation of crop areas.

Among the three optimization scenarios, the seasonal and season-spatial optimization scenarios only utilize the NDR model and the nonlinear programming model, while integrated optimization scenario requires all three models including the land use model.

## InVEST NDR model (Step 1)

InVEST (Integrated Valuation of Ecosystem Services and Tradeoffs) is a suite of spatially explicit models for evaluating trade-offs associated with land use induced ecosystem service changes, developed by the partners of the Natural Capital Project (https://naturalcapitalproject.stanford.edu/software/invest). The NDR model simulates nitrogen movement across space, taking into account both abiotic and biotic factors. We extend the literature on applications of the NDR model by assessing nitrogen retention and delivery by pixel by season at national level, and map nitrogen sources from districts and their transport to waterbodies.

The model first estimates the pixel-level total nitrogen load, nitrogen retained by vegetation and topographic features, and nitrogen delivered to the water outlet for each season, which are then aggregated to the district level. The main characteristics of the model include the following. First, surface and subsurface flow paths are identified from the digital elevation model (DEM), and the nitrogen load is defined mainly as nitrogen application of rice land and non-rice land with minor input from natural causes. Soil and nutrient retention parameters are mapped to the land use types and used to simulate the transport process. Second, the loads are routed along topographically defined flow paths, with a proportion of the load being removed in each cell. At the watershed or sub watershed outlet, nitrogen export is computed as the sum of the pixel-level contributions.

There are two sources of nitrogen loads. One is from fertilizer application of cropland and the other is from natural resources, such as rainfall. Since we focus on the first one, it is necessary to exclude the nitrogen from natural resources while modeling the delivery ratio. We run two iterations of the NDR model. First, nitrogen loads from both are used in the simulation. In the second iteration, the fertilizer application in the cropland is set to 0, so all the nitrogen load is from natural resources. The difference of nutrient loads and nitrogen exports between the two iterations is calculated, and nitrogen delivery ratios of the fertilizer application are the total nitrogen exported from the district divided by the nitrogen load from the district (see Supplementary Materials “NLP_parameter.xlsx-ndr sheet”).

The NDR model uses three types of input data: the land cover and land use (LCLU) maps, georeferenced attributes at various spatial resolution, and tabular datasets. The spatial information is used to evaluate the ecosystem services of nutrient retention by vegetation coverage and their changes. The LCLU map of Bangladesh in 2015 was developed by Center for Spatial information Science and Systems, George Mason University (http://cloud.csiss.gmu.edu/ganges-lulc/) using time series of Landsat scenes from NASA [5]. The datasets map major land cover types at 30-meter grid. The major land cover types include cropland, forest, artificial surfaces, water, grassland, bareland, and wetland. It provides the baseline of land characteristics and served as one of the key input layers to the model.

Geospatial attribute datasets include two categories: One of them is from existing datasets such as DEM and precipitation. The elevation from WorldClim is used to describe the topology and drive the DEM which generate the stream flow maps with hydrographic maps of the country [6]. The DEM also defines flow routing and directions between pixels. The monthly rainfall data from Climate Hazards group InfraRed Precipitation with Stations (CHIRPS) are used to capture the rainfall variation across both spatial and temporal in the country [7]. The other type of spatial data is district-level fertilizer application data of cropland derived from the Bangladesh Integrated Household Survey (BIHS) [8]; the fertilizer data are then georeferenced so they could be overlaid with other spatial datasets including land cover and rainfalls. The tabular data are drawn from the literature and existing database from the InVEST model. The parameters include nitrogen load for each none-cultivated land types. The soil nitrogen retention efficiency adopted from the harmonized world soil database [9]. The additional subsurface parameters such as critical distance value for subsurface transport of nitrogen (constant over the landscape) and subsurface retention efficiency for nitrogen (constant over the landscape) are not site specific and are constant over landscape. Pärn et al. [10] provides average values for the partitioning of nitrogen loads between leaching and surface runoff. From Mayer et al. [11], a global average of 200-meter for the retention length and 80% for retention efficiency can be assumed for vegetated buffers. All the input data of the NDR model can directly be downloaded from https://www.dropbox.com/sh/z7adri81c8lqdoq/AABDxkthW1ieoTX95BBEaZAJa?dl=0.

## Land use model (Step 2)

Consider a farm that has $\bar{L}$ acres of cropland. Let *r* denote three cropping seasons: spring, summer, and winter, indexed by 1, 2, and 3, respectively. Let *j* denote three types of cropland use: rice, non-rice crops, and idle, indexed by 1, 2, and 0, respectively. Let $\pi (p_{rj},L_{rj})$ be the profit function for crop j in season *r*, where $p_{r}=\left(p_{r1},p_{r2},0\right)$ is exogenous net prices for rice, non-rice crops, and idle use the farmer faces, and $L_{rj}$ is the amount of land the farmer chooses for land use j in season *r*. The farmer's objective is to choose the land allocation that maximizes total profit

(1) $$\max_{\left\{L_{rj}\right\}}\sum_{r=1}^{3}\sum_{j=0}^{2}\pi \left(p_{rj},L_{rj}\right),$$

subject to

(2) $$\sum_{j=0}^{2}L_{rj}=\bar{L},\;\text{for}\;r\;=\;1,\;2,\;3.$$

The solution to this problem gives the optimal land allocation $L_{rj}^{*}=L_{rj}\left(p_{r},\bar{L}\right)$. Assume that the function $L_{rj}\left(p,\bar{L}\right)$ is homogeneous of degree one in $\bar{L}$. Then

(3) $$L_{rj}^{*}=L_{rj}\left(p_{r},\bar{L}\right)=L_{rj}\left(p_{r},1\right)\bar{L},\;\forall \;r,j.$$

Eq. (3) can be written in share form as

(4) $$s_{rj}^{*}\equiv \frac{L_{rj}^{*}}{\bar{L}}=L_{rj}\left(p_{r},1\right),\;\forall \;r,j.$$

The optimal land allocation in reason *r* depends on the net prices for rice and non-rice crops in that season. The system of share equations (4) serves as the theoretical basis for the development of an empirical land use model. The model treats temporarily idle cropland as a residual choice and therefore captures the evolution of cropping intensity in response to changes in seasonal crop net price. Unlike the intra-seasonal allocation, it is not conceptually clear how crop allocation correlates across seasons. This correlation will be captured in the empirical estimation.

A common practice in the land use modeling literature is to assume that the share equations take the logistic form, which will guarantee that the predicted shares lie within a zero-one interval. But in this study, the presence of inter-seasonal correlation prohibits the use of standard logistic regression to estimate Eq. (4). While a multivariate probit model allows for the joint estimation of multiple correlated outcomes, it works only for binary choices, rather than for continuous outcomes as in this study. To overcome this estimation challenge, we take advantage of the generalized linear property of logistic form and converts the nonlinear share equations to a system of linear equations.

Specifically, assume that the share equations take the logistic form:

(5) $$s_{rj}^{*}=\frac{\text{exp}\left(s_{rj}^{0}\alpha_{r}+x_{rj}\beta_{rj}+z_{r}\gamma_{rj}\right)}{\sum_{k=0}^{2}\text{exp}\left(s_{rk}^{0}\alpha_{r}+x_{rk}\beta_{rk}+z_{r}\gamma_{rk}\right)},\;\forall \;r,j,$$

where exp(·) is the exponential function. The term $s_{rj}^{0}$ represents a lagged crop share in the previous period—an inertia variable which captures the incentive-based variables such as net price, market accessibility, and land use conversion costs as well as the location-specific characteristics such as land suitability, soil properties, topography, and weather conditions. Including an inertia variable is an empirical strategy to absorb unobserved factors (e.g., agroecological conditions and land use conversion costs) affecting land use choice and is vital to model agricultural land use. Since $s_{rj}^{0}$ has captured the net price for choice *j* for the previous period, $p_{rj}$ is replaced with $x_{rj}$, representing the change in net price from the previous period to the current period. Analogously, the term $z_{r}$ represents a vector of changes in other time-varying variables that may potentially drive land use change and $z_{r}$ = (*change in road density, change in seasonal precipitation*). Road density measures market accessibility, which is important to transport perishable crops such as vegetables to a market. Precipitation change has different implications in different seasons. High rainfall may increase the risk of floods and crop failure in monsoon seasons but helps maintain soil moisture thereby reducing irrigation cost in dry seasons. The terms $\alpha_{r}$, $\beta_{rj}$, and $\gamma_{rj}$ are coefficient parameters on $s_{rj}^{0}$, $x_{rj}$, and $z_{r}$.

Let the idle use be a residual category (*j* = 0) and define $\eta_{rj}\equiv \gamma_{rj}-\gamma_{r0}$. Eq. (5) implies that

(6) $$\ln\left(s_{rj}^{*}/s_{r0}^{*}\right)=\left(s_{rj}^{0}-s_{r0}^{0}\right)\alpha_{r}+x_{rj}\beta_{rj}+z_{r}\eta_{rj},\;\text{for}\;r\;=1,\;2,\;3\;\text{and}\;j\;=\;1,\;2.$$

By taking the logarithm of *the odds of crop j against idle*, the logistic form is transformed to a set of six linear equations. The method of seemingly unrelated regression (SUR) is applied to estimate the systems of equations (6) using the cross-sectional district-level data. The SUR method is a generalization of ordinary least squares (OLS) for multi-equation systems and allows the correlation among the errors in different equations to improve the regression estimates [12]. Admittedly, this estimation approach will not, in general, be fully efficient, but its great advantage lies in its ability to retain the important feature of the share equations for intra-season allocation while allowing for inter-season correlation of land use decision. Such an estimation strategy has been applied by political scientists to model election returns in multiparty elections [13]. The SUR model can be estimated with any standard statistical software such as SAS, R, or STATA.

The estimated econometric land use model relies on district-level agricultural statistics for two years, 2013 and 2017 [14,15]. For each year, we use the area of physical cropland and season-wise planting area to calculate land use shares of rice, non-rice crops, and those left unsown (hereafter idle) for spring, summer, and winter, respectively. The production statistics, in combination with the district-level farmgate prices and production costs associated with labor, seeds, irrigation, fertilizer, and other tools collected from BIHS [8] are used to calculate district-level net price (i.e., price minus cost) for rice and non-rice crops each season. Road density is calculated as the total length of paved roads in a district divided by the land area of that district, collected from agricultural statistics [[14], [15]]. For each season, we calculate the average monthly precipitation during planting period, where rainfall data are collected from CHIRPS [7]. Outputs from the land use model include a system of six equations that establishes the relationship of rice and non-rice crop shares and the aforementioned factors for each season (Table 1).

**Table 1:** Estimated Land Use Model for 2017 Crop Allocation Using the SUR Method.

	Spring		Summer		Winter	
	Rice	Non-rice	Rice	Non-rice	Rice	Non-rice
	(1)	(2)	(3)	(4)	(5)	(6)
Net price change	-0.066	0.130^⁎⁎^	0.065^⁎⁎^	0.027	0.419^⁎⁎⁎^	0.328^⁎⁎⁎^
	(0.056)	(0.057)	(0.031)	(0.070)	(0.116)	(0.119)
Road density change	-0.01	0.46	6.99^⁎⁎^	8.40^⁎⁎⁎^	-2.59	-1.59
	(1.94)	(1.04)	(2.72)	(3.06)	(2.97)	(3.33)
Precipitation change	0.21	0.29^⁎⁎⁎^	-0.07	-0.10	2.72	13.38^⁎⁎⁎^
	(0.20)	(0.10)	(0.22)	(0.24)	(2.88)	(2.25)
Difference in initial share	3.75^⁎⁎⁎^	3.75^⁎⁎⁎^	2.68^⁎⁎⁎^	2.68^⁎⁎⁎^	5.15^⁎⁎⁎^	5.15^⁎⁎⁎^
	(0.28)	(0.28)	(0.37)	(0.37)	(0.47)	(0.47)
System Weighted *R*-squared	0.788					

Note: The sample includes 64 districts. Standard error is in parentheses. *, **, and *** indicate statistical significance at the 10, 5, and 1% levels, respectively.

## Nonlinear programming model (Step 3)

### Modeling setup

The optimization problem presents an objective of minimizing total annual nitrogen export aggregated across districts and seasons, subject to a set of economic and physical constraints concerning total annual output value and cropland availability. The model can be solved using optimization software such as GAMS, MATLAB, and SAS® Optimization.

Assume that the central government is concerned about national agricultural runoff pollution and attempts to minimize the total nitrogen export while maintaining the value of total crop production and guaranteeing food security in the country. Let D be the total number of districts (there are 64 districts in total), the government's problem can be written as

(7a) $$\min_{\left\{N_{irj},L_{irj}\right\}}\sum_{i=1}^{D}\sum_{r=1}^{3}\sum_{j=1}^{2}\theta_{ir}N_{irj}L_{irj}$$

(7b) $$s.t.\;\sum_{i=1}^{D}\sum_{r=1}^{3}\sum_{j=1}^{2}L_{irj}y_{irj}\left(N_{irj}\right)p_{irj}\geq v^{b}$$

(7c) $$\sum_{i=1}^{D}\sum_{r=1}^{3}L_{ir1}y_{ir1}\left(N_{ir1}\right)\geq Y_{1}^{b}$$

(7d) $$\sum_{j=1}^{2}L_{irj}\leq {\bar{L}}_{ir},\;i=1,\ldots ,\;D;\;r=1,\;2,\;3$$

(7e) $$\sum_{r=1}^{3}\sum_{j=1}^{2}L_{irj}\leq CI_{i}{\bar{L}}_{i},\;i=1,\ldots ,\;D,$$

where $L_{irj}$ represents the total harvested area of crop j in season r, district i, and $N_{irj}$ represents the associated per acre nitrogen input; $L_{irj}$ and $N_{irj}$ are decision variables that the government jointly controls. The parameter $\theta_{ir}$ is a district-specific, season-wise ratio of the nitrogen export to the nitrogen input. A linear relationship is assumed between the export and the input and this relationship varies by season and over space. Therefore, the term $\theta_{ir}N_{irj}L_{irj}$ in the objective function (7a) represents the nitrogen export from growing crop j in season r, district i.

The economic constraint in (7b) states that the farmers as a whole would not be worse off from the reallocation of crop production and nitrogen application in order to minimize agricultural runoff pollution. The parameter $p_{irj}$ represents the price of crop j grown in season r, district i. The parameter $v^{b}$ is the value of total agricultural production in the baseline. It is assumed that crop yield $y_{irj}$ is a function of nitrogen input, denoted as $y_{irj}\left(N_{irj}\right)$, where technology and other inputs are implicitly captured in the function.

The food security constraint in (7c) states that the country's total rice production would be maintained at least at the baseline level, denoted as $Y_{1}^{b}$. As the dominant food crop of Bangladesh, rice provides about two-third of total calorie supply and about one-half of the total protein intake of an average person in the country. This constraint indicates that national food security is not compromised as a result of the environmental initiative.

The constraints in (7d) and (7e) are physical constraints, where ${\bar{L}}_{ir}$ is the area of cropland potential in season r, district i; ${\bar{L}}_{i}$ is the area of cropland available in district i; $CI_{i}$ is the annual cropping intensity in district i under the baseline. Constraint (7d) states that in each season the total cropping area cannot exceed the land area potentially suitable for crop production. Constraint (7e) states that the annual harvested area in each district cannot exceed the baseline annual harvested area at the current cropping intensity.

For the third optimization scenario, integrated optimization, we need to embed the econometric land use model in the optimization problem. Of particular interest is how the government can use price instruments to guide the reallocation of crop production to balance food security and environmental sustainability. Ideally, one may combine the two models by directly inserting the land use share Eq. (5) into equations (7a)‒(7e) and solving for the optimal per-acre nitrogen input and net prices. But such an integration involves a computational challenge because exponential functions appear in both the denominator and the numerator. An alternative strategy is to calculate the total derivative of the share equation with respect to the net prices at the baseline level and write the share equation as a function of the marginal effects of net prices. This linear approximation greatly reduces the computational burden and will be adopted here.

Specifically, the decision variable $L_{irj}$ is replaced in optimization (7a)‒(7e) by $L_{irj}\left(x_{ir1},x_{ir2}\right)$, which represents the cropping area equation with two unknown arguments $x_{ir1}$ and $x_{ir2}$— the change in the net price of rice and non-rice crops from the baseline. With this substitution, $N_{ir}$, $x_{ir1}$, and $x_{ir2}$ are decision variables that the government jointly controls. From Eq. (5), one can derive the marginal effect of $x_{rk}$ on crop share $s_{rj}$, denoted as $\frac{\partial s_{rj}}{\partial x_{rk}}$, and write the land equation as

(8) $$L_{irj}\left(x_{ir1},x_{ir2}\right)={\bar{L}}_{i}\left(s_{irj}^{b}+\frac{\partial s_{rj}}{\partial x_{r1}}{|}_{i}x_{ir1}+\frac{\partial s_{rj}}{\partial x_{r2}}{|}_{i}x_{ir2}\right),$$

where $s_{irj}^{b}$ is the crop share in the baseline; the last two terms in the parentheses represent changes in the share of crop j due to price change. The marginal effects $\frac{\partial s_{rj}}{\partial x_{rk}}$ (k=1,2) are evaluated at every district-level baseline observation i,

(9) $$\frac{\partial s_{rj}}{\partial x_{rk}}{|}_{i}=s_{irj}^{b}\left(I_{\left(j=k\right)}-s_{irk}^{b}\right){\hat{\beta }}_{rk},\;i=1,\ldots ,\;D;\;r=1,\;2,\;3;\;j=1,2;\;k=1,2,$$

where $I_{\left(j=k\right)}$ is a binary variable indicating whether or not j=k; ${\hat{\beta }}_{rk}$ is the estimated coefficient of the net price change $x_{rk}$ for crop k in season r. The point estimate ${\hat{\beta }}_{rk}$ will be assigned zero when it is statistically insignificant at the 10% level.

Furthermore, the net price change is accounted for when evaluating the economic constraint (7b), which is subsequently replaced by the following constraint:

(10) $$\sum_{i=1}^{D}\sum_{r=1}^{3}\sum_{j=1}^{2}L_{irj}\left(x_{ir1},x_{ir2}\right)y_{irj}\left(N_{irj}\right)\left[p_{irj}+x_{irj}+\Delta c_{irj}\left(N_{irj}\right)\right]\geq v^{b},$$

where $\Delta c_{irj}\left(\cdot \right)$ is the change in average production cost associated with nitrogen application and is calculated as

(11) $$\Delta c_{\mathrm{irj}}\left(N_{\mathrm{irj}}\right)=\frac{p_{\mathrm{ir}}^{N}N_{\mathrm{irj}}}{y_{\mathrm{irj}}\left(N_{\mathrm{irj}}\right)}-\frac{p_{\mathrm{ir}}^{N}N_{\mathrm{irj}}^{b}}{{\underset{...}{y_{\mathrm{irj}}\left(N_{\mathrm{irj}}^{b}\right)}}_{c_{\mathrm{irj}}^{b}}}=\frac{p_{\mathrm{ir}}^{N}N_{\mathrm{irj}}}{y_{\mathrm{irj}}\left(N_{\mathrm{irj}}\right)}-c_{\mathrm{irj}}^{b},$$

where $N_{irj}^{b}$ is the baseline per acre nitrogen input, $p_{ir}^{N}$ is the price of nitrogen inputs adjusted according to the price of urea, and $c_{irj}^{b}\equiv \frac{p_{ir}^{N}N_{irj}^{b}}{y_{irj}\left(N_{irj}^{b}\right)}$, representing the baseline average production cost associated with nitrogen application. While that price is relatively stable across seasons, $p_{ir}^{N}$ is allowed to vary by season because of the seasonal variation in the relationship of nitrogen inputs and urea application. By definition, $x_{irj}$ consists of the changes in both crop price and average production cost. The change in crop price is given by $x_{irj}+\Delta c_{irj}\left(N_{irj}\right)$ and the new crop price equals $p_{irj}+x_{irj}+\Delta c_{irj}\left(N_{irj}\right)$.

### Parameterization

The nitrogen export-input ratio $\theta_{ir}$ was calculated from the outputs of the InVEST-NDR model. The baseline value of total agricultural production $v^{b}$ (*=* 1,410.8 billion Taka), total rice production $Y_{1}^{b}$ (*=* 35.9 million metric ton), the district-level physical cropland area ${\bar{L}}_{i}$, annual cropping intensity $CI_{i}$, agricultural production value $v_{i}^{b}$, and the district- and season-wise crop shares $s_{irj}^{b}$ are collected from agricultural statistics and BIHS [8,15], the same sources of data used in the land use model. The baseline district-level seasonal crop prices $p_{irj}$ are extrapolated by combining the national-level annual price data from agricultural statistics [15] and the household-level price data for 2014 from BIHS [8], assuming a constant price growth rate across the country and being averaged between 2016 and 2017.

The area of cropland potential ${\bar{L}}_{ir}$ is the product of ${\bar{L}}_{i}$ and the agroecological-zone-based seasonal cropping intensity potential $CI_{ir}^{p}$. Bangladesh's division into 30 agroecological zones (AEZs) is based on hydrology, physiography, soil type, tidal activity, cropping pattern, and season. An AEZ indicates an area characterized by homogeneous agricultural and ecological characteristics. If an AEZ comprises multiple districts, $CI_{ir}^{p}$ is the maximum ratio of seasonal cultivated area to ${\bar{L}}_{i}$ (i.e., the ratio of the “frontier” district(s)) and remains the same across districts. If a district is split up into multiple AEZs, $CI_{ir}^{p}$ is the area-based weighted average of the maximum ratios.

The yield function is assumed to take the Cobb-Douglas functional form with nitrogen (urea) input $y_{irj}=\delta_{irj}N_{irj}^{\rho_{rj}}$, leaving all other inputs to be captured in the unknown coefficient $\delta_{irj}$ that represents total factor productivity. For the group of non-rice crops, $y_{irj}$ is the area-based weighted average rice-equivalent yield of all non-rice crops in district i season r, where rice-equivalent yield converts the yield of non-rice crops into equivalent rice yield on a price basis. Of particular interest is the yield-nitrogen elasticities $\rho_{rj}$, which are estimated using the household-level data from BIHS [8]. Specifically, we adopt a two-step transformation. First, we transform the nonlinear Cobb-Douglas function to a linear equation by taking logarithms on both sides. Second, we replace the logarithm transformation with the inverse hyperbolic sine transformation; such a transformation gives results identical to using the logarithm for non-zero observations while also being able to handle the zeroes with no crude transformation of the data. We fit the cross-sectional household data into the following equation

(12) $$\sinh^{-1}y_{hrj}=\rho_{rj}\sinh^{-1}Urea_{hrj}+b_{rj}^{1}\sinh^{-1}Labor_{hrj}+b_{rj}^{2}Hybrid_{hrj}+b_{rj}^{3}HYV_{hrj}+\varepsilon_{hrj},\;\text{for}\;r=1,\;2,\;3\;\text{and}\;j=1,\;2.$$

The subscript h indexes household; other subscripts are consistent with the definitions in the article. The term $y_{hrj}$ represents the yield of crop j in season r in a household; $Urea_{hrj}$ represents the associated per acre urea application rates; $Labor_{hrj}$ represents the corresponding per acre labor hours; $Hybrid_{hrj}$ and $HYV_{hrj}$ are the share of cultivated area of hybrid crops and high-yielding varieties (HYV) crops, respectively. The coefficients $\rho_{rj}$, $b_{rj}^{1}$, $b_{rj}^{2}$, and $b_{rj}^{3}$ are unknow parameters to be estimated. The stochastic error term $\varepsilon_{hrj}=District_{i}+\epsilon_{hrj}$; $\varepsilon_{hrj}$ contains district fixed effects, $District_{i}$, to absorb district-specific unobserved factors. We estimate Eq. (12) for each of two crop groups in each season, separately (Table 2). Given the estimated elasticities ${\hat{\rho }}_{rj}$, the coefficient $\delta_{irj}$ is calculated in the following calibration process

(13) $${\hat{\delta }}_{\mathrm{irj}}={\frac{y_{\mathrm{irj}}}{N}}_{\mathrm{irj}}^{{\hat{\rho }}_{\mathrm{rj}}}\},\mathrm{for}\;i=1,\cdots ,\;D;\;r=1,\;2,\;3;\;j=1,\;2.$$

**Table 2:** Estimated Yield Function by Crop and Season Using the Household-level Data.

	Spring		Summer		Winter	
	Rice	Non-rice	Rice	Non-rice	Rice	Non-rice
	(1)	(2)	(3)	(4)	(5)	(6)
Urea (nitrogen)	0.110^⁎⁎^	0.079^⁎⁎⁎^	0.120^⁎⁎⁎^	0.109^⁎⁎^	0.045^⁎⁎⁎^	0.118^⁎⁎⁎^
Labor	0.571^⁎⁎⁎^	0.559^⁎⁎⁎^	0.755^⁎⁎⁎^	0.615^⁎⁎⁎^	0.102^⁎⁎⁎^	0.457^⁎⁎⁎^
Share of hybrid	0.409	0.114	0.700^⁎⁎⁎^	-0.616^⁎⁎^	0.176^⁎⁎⁎^	0.328^⁎⁎⁎^
Share of HYV	0.171	-0.001	0.492^⁎⁎⁎^	0.404^⁎⁎^	-	0.131^⁎⁎^
District fixed effects	Yes	Yes	Yes	Yes	Yes	Yes
*R*-squared	0.530	0.287	0.354	0.464	0.136	0.482
No. of observations	315	689	2323	285	2103	1429

Note: The arcsinh transformation is applied to both the dependent variable and urea and labor inputs. The estimate of *share of HYV* in col. 5 is missing because all winter rice is either hybrid or HYV (no local varieties), producing a perfect collinearity issue. *, **, and *** indicate statistical significance at the 10, 5, and 1% levels, respectively.

## Validation

Goodness of fit of the econometric land use model and the yield function is provided in Supplementary Materials “LandUseModel.xlsx” and “YieldFunction.xlsx”. It is important to note that direct validation of large-scale soil/water nutrient models remains challenging largely because of the prohibitively high cost associated with soil/water sampling and laboratory testing. For Bangladesh, a central database of water sampling data is not available. Yet this study relies on BIHS to acquire nitrogen fertilizer application, the most important input data required by the NDR. BIHS is the most comprehensive, nationally representative survey in Bangladesh. Other NDR input parameters were acquired from sources published at the Natural Capital project (https://naturalcapitalproject.stanford.edu/software/invest). The database from the Natural Capital project is developed from over 200 peer-reviewed publications in agronomy, hydrology, and conservation agriculture. Moreover, the NDR model and its applications have been published in peer-reviewed studies. Despite thin studies on nitrogen pollution or agricultural runoff in Bangladesh, the smaller scale study by Rahaman et al. [16] reveals similar seasonal and spatial pattern for the coastal region. Specifically, Rahaman et al. [16] collected water samples of the three major river systems of Bangladesh in summer and winter seasons from several sampling stations located in three districts—Satkhira, Khulna, and Bagerhat. They find a steadily higher nitrogen concentration in winter across all sampling stations, which is consistent with our findings, i.e., the per-acre nitrogen export is higher in winter than summer in the three districts (Table 3). Spatially, both Rahaman et al. [16] and our study find that summer nitrogen runoff is lowest in Khulna and winter nitrogen runoff is lowest in Bagerhat. As for the spatial comparison of other districts, our finding does not conform to Rahaman et al.’s [16]. This inconsistency is partly because the three districts are adjacent to each other and some water samples of different districts were actually collected from the same river (i.e., a river crosses the political border of two adjacent districts). Thus, seasonal comparison is more meaningful than spatial comparison. Optimization results are provided in Supplementary Materials “NLP_output.xlsx”.

**Table 3:** A Comparison of Nitrogen Pollution between Rahaman et al. (2013) and This Study.

	Rahaman et al. (2013)			This study	
River System	Sampling location (district)	Sampling season	Total nitrogen concentration (mg/L)	Nitrogen export (kg/acre)	Nitrogen delivery ratio
Rupsha-Passur River	Khulna	Summer	2.10±0.41	0.153	0.15
Rupsha-Passur River	Khulna	Winter	4.47±0.72	1.795	0.27
Arpangashia-Malancha River	Satkhira	Summer	2.78±0.38	0.706	0.12
Arpangashia-Malancha River	Satkhira	Winter	4.22±1.05	2.381	0.26
Baleswar-Bhola River	Bagerhat	Summer	3.02±0.43	0.379	0.11
Baleswar-Bhola River	Bagerhat	Winter	3.87±1.20	0.625	0.20

Note: Satkhira, Khulna, and Bagerhat are three districts adjacent to each other and some water samples of different districts were actually collected from the same river (i.e., a river crosses the political border of two adjacent districts). Thus, the seasonal comparison is more meaningful than the spatial comparison.

## Ethics statements

Our work did not involve human subjects, animal experiments, or social media platforms

## CRediT author statement

**Man Li:** Conception and design of the study, Methodology, Software, Validation, Writing-Original draft, Writing-Reviewing and Editing

**Zhe Guo:** Conception and design of the study, Methodology, Software, Validation, Writing-Original draft, Writing-Reviewing and Editing

**Wei Zhang:** Conception and design of the study, Methodology, Validation, Writing-Original draft, Writing-Reviewing and Editing

## Declaration of Competing Interest

The authors declare that they have no known competing financial interests or personal relationships that could have appeared to influence the work reported in this paper.

## Data Availability

Data will be made available on request.

## Appendix Supplementary materials

Image, application 1

Image, application 2

Image, application 3

Image, application 4

Image, application 5
